# A Risk Score to Identify Low Bone Mineral Density for Age in Young Patients with Anorexia Nervosa

**DOI:** 10.3390/nu17010161

**Published:** 2024-12-31

**Authors:** Laurent Maimoun, Helena Huguet, Eric Renard, Patrick Lefebvre, Maude Seneque, Laura Gaspari, Vincent Boudousq, Lisa Maimoun Nande, Philippe Courtet, Charles Sultan, Denis Mariano-Goulart, Marie-Christine Picot, Sebastien Guillaume

**Affiliations:** 1 Physiology and Experimental Medicine of the Heart and Muscles (PhyMedExp), CNRS, INSERM, University of Montpellier, 34295 Montpellier, France; d-mariano_goulart@chu-montpellier.fr; 2 Department of Nuclear Medicine, CHU Montpellier, 34295 Montpellier, France; 3 Service de Médecine Nucléaire, Hôpital Lapeyronie, 371, Avenue du Doyen Gaston Giraud, CHU de Montpellier, CEDEX 5, 34295 Montpellier, France; 4 Unit of Clinical Research and Epidemiology, CHU Montpellier, University of Montpellier, 34000 Montpellier, France; h-huguet@chu-montpellier.fr (H.H.); mc-picot@chu-montpellier.fr (M.-C.P.); 5 Department of Endocrinology, Diabetes, Nutrition, CHU Montpellier, 34295 Montpellier, France; e-renard@chu-montpellier.fr (E.R.); p-lefebvre@chu-montpellier.fr (P.L.); 6 Central Information Commission (CIC), INSERM 1411, 34295 Montpellier, France; 7 Institute of Functional Genomics, CNRS, INSERM, University of Montpellier, 34295 Montpellier, France; 8 Department of Emergency and Post-Emergency Psychiatry, CHU Montpellier, INSERM, University of Montpellier, 34295 Montpellier, France; maude.seneque@gmail.com (M.S.); p-courtet@chu-montpellier.fr (P.C.); s-guillaume@chu-montpellier.fr (S.G.); 9 Unit of Paediatric Endocrinology and Gynaecology, CHU Montpellier, University of Montpellier, 34295 Montpellier, France; laura.gasparisultan@gmail.com (L.G.); pr.charles.sultan@gmail.com (C.S.); 10 Department of Nuclear Medicine, CHU Nîmes, 30029 Nîmes, France; vincent.boudousq@chu-nimes.fr; 11 Departement of Biophysique, Faculty of Medicine, University of Montpellier, 34295 Montpellier, France; lisa.maimounnande@gmail.com

**Keywords:** anorexia nervosa, bone demineralization, low areal bone mineral density, risk score

## Abstract

Objective: Developing a scoring assessment tools for the determination of low bone mass for age at lumbar spine and hip in patients with anorexia nervosa (AN). Methods: The areal bone mineral density (aBMD) was determined with dual-energy X-ray absorptiometry (DXA). In 331 women with AN and 121 controls, aged from 14.5 to 34.9 years, univariate and multivariate logistic regression analyses were performed to address the association of Z-score aBMD evaluated at lumbar spine and hip with several parameters. Results: For the lumbar spine and hip, the three risk factors significantly and independently associated with Z-score aBMD were age of patients (variable in class ≥20 year vs. <20 year), minimal disease-related BMI (continuous variable), and duration of amenorrhea without contraceptive use (variable in class ≥18 months vs. <18 months), with close values for the odds ratio for the two bone sites. A simple risk score equation was developed and tested combining only these three parameters. The AUC’s measuring the score’s performance were, respectively, 0.85 [95% CI: 0.79–0.90] with a sensitivity of 83% and specificity of 71%, and 0.82 [95% CI: 0.76–0.86] with a sensitivity of 92% and specificity of 55% to detect low aBMD in lumbar spine and hip. The cut-off values for low bone mass for age were 0.9 and 1.33 for the two bone sites. The prediction model revealed that a minimum of 83% of the patients presenting low bone mass for age were correctly identified. Conclusions: the study presents for the first time a risk score for diagnosing low bone mass for age in young patients with AN. Considering its excellent sensitivity, and its ease of use, requiring only three parameters that are well identified in this disease, this new score may be useful in clinical settings when DXA scans are not feasible.

## 1. Introduction

It is widely acknowledged that anorexia nervosa (AN) induces early and severe bone loss [1,2,3]. For example, we reported that 2 years after AN onset, the areal bone mineral density (aBMD) was reduced by 15% at trabecular (lumbar spine) and cortical (femur) sites [3]. Moreover, it was demonstrated that 92% and 38% of women with AN develop, respectively, osteopenia and osteoporosis, as defined by the WHO [4]. The combination of bone mass loss and microarchitecture alterations [5,6] is the origin of the high risk of fragility fractures [7,8]. Consequently, systematic, adapted, and earl, bone mass monitoring should be instituted for these patients to reduce the impact on peak bone mass acquisition and the subsequent risk of osteoporosis [3].

In the general population, dual-energy X-ray absorptiometry (DXA) is the gold standard for diagnosing osteoporosis [9], and it is used for several indications in adults [10]. In France, in patients with AN, it is recommended that the first DXA measures be performed after 6 months of amenorrhea, with monitoring every 2 years for those with low aBMD and persistent amenorrhea [11]. However, accessibility to this technique may be limited, as patients are often admitted to psychiatric residential treatment centers that are not authorized to use this X-ray device. A new approach to screening most of these patients could be based on a scoring tool to assess the risk of low aBMD for age constructed from knowledge on this specific type of bone loss. For example, clinical and disease-related factors such as current weight, lean tissue mass, body mass index (BMI), age of AN onset, and minimal disease-related BMI are positively correlated with both aBMD and microarchitecture parameters, whereas the disease and amenorrhea durations exert negative influences and may be considered to have deleterious effects [2,3,4,5,12,13,14,15,16,17,18,19]. Moreover, we more recently observed that the major independent factor associated with reduced aBMD is the minimal disease-related BMI, whereas biological factors like bone remodeling markers and periostin are not strongly associated [20]. These results indicate that the younger the patient is at the beginning of the disease and the longer the disease has lasted, the more severe the impact on bone tissue will be and the more difficult it will be to restore normal bone condition. However, we must keep in mind that the relationship between these factors and aBMD may be modulated by external factors such as oral contraceptive (OC) use [21].

The first aim of this study was to identify the main clinical and disease-related factors associated with aBMD in a large group of well-characterized young patients with AN. As the influence of these factors may vary according to the bone site evaluated [22], two distinct analyses were performed for lumbar spine and total hip. The second aim was to build a scoring assessment tool from the factors most associated with aBMD in order to determine the normal and low aBMD for age in this specific population of young patients. We hypothesized that the lowest disease-related BMI, duration of amenorrhea, and duration of AN are the main clinical factors related to bone demineralization and that the combination of these factors could lead to the creation of a risk score that is sufficiently sensitive and specific to be clinically useful.

## 2. Materials and Methods

### 2.1. Study Design

This study followed a case–control design. Study approval was obtained from the Regional Research Ethics Committee (Comité de Protection des Personnes Sud Mediterranee IV, Montpellier, France; reference: 11 02 03; date 5 June 2012), and permission for the clinical trials was granted by the French Agency for the Safety of Health Products (Agence Française de Sécurité Sanitaire des Produits de Santé; AFSSAPS; reference: 2011-A00108-33). Written informed consent was obtained from all participants, or their parents when the volunteers were minors.

### 2.2. Subjects

A total of 434 adolescents and young women were enrolled in this study, 313 in the case group and 121 in the control group. None were taking medications known to affect bone metabolism, including glucocorticoids (>3 months), bisphosphonates, selective estrogen receptor modulators, teriparatide, and denosumab, and none presented with primary amenorrhea.

Patients with AN were recruited from the Department of Endocrinology (CHU Montpellier) between 2009 and 2016 and fulfilled the criteria for the diagnosis of restrictive AN according to DSM V (Diagnostic and Statistical Manual of Mental Disorders V) [23]. A full description of the diagnostic procedure can be found elsewhere [24].

An age-matched control group that consisted of healthy normal-weight adolescents and young women (18 < BMI < 25 kg/m^2^) was also recruited in the community by advertisement. None had a history of eating disorders or other psychiatric illness, as determined by Mini International Neuropsychiatric Interview [25] and the SCOFF questionnaire [26]. None of the controls presented a menstrual cycle disorder.

### 2.3. Data Collection

#### 2.3.1. Anthropometric Data

Standing height was measured with a stadiometer to the nearest 0.1 cm. Weight was determined using a weight scale with a precision of 0.1 kg. BMI was calculated as weight (kilograms) divided by the square of height (meters). The weight standard deviation score (weight SDS) and height standard deviation score (height SDS) were determined according to the French standard curves.

#### 2.3.2. Medical and Menstrual Histories

Each subject or her parents responded to a medical questionnaire designed to assess the general medical and menstrual history, with questions on the age of menarche, the presence of menstrual disorders, and the use of oral contraceptives (OCs). Moreover, age at disease onset, the duration of the eating disorder, and body weight variation were also collected.

#### 2.3.3. Bone Mineral Density, Body Fat, and Fat-Free Soft Tissues

DXA (Hologic QDR-4500A, Hologic, Inc., Waltham, MA, USA) was used to measure the areal bone mineral density (aBMD; g/cm^2^) of the whole body and at r lumbar spine (L1–L4), the proximal part of the hip, and the dominant arm radius. The soft tissue body composition (fat-free soft tissue [FFST, kg] and fat mass [FM, kg and %]) was derived from the whole-body scan. All scanning and analyses were performed by the same operator to ensure consistency, after following standard quality control procedures. Quality control was checked daily by scanning a lumbar spine phantom (DPA/QDR-1; Hologic x-calibre anthropometric spine phantom). The coefficient of variation (CV) given by the manufacturer was 0.8% for spine, 1.1% at the hip, 0.8% at the radius, and <1% for FFST and FM.

#### 2.3.4. Classification of Patients According to aBMD Values

The International Society for Clinical Densitometry (ISCD) recommends in females prior to menopause to use Z-scores and not T-scores. Patients with a Z-score of −2.0 or lower are defined as “below the expected range for age” and considered in the current study as the “low aBMD group”, while those with a Z-score above −2.0 aer “within the expected range for age” and considered as the “normal aBMD group” [27,28].

### 2.4. Statistical Analysis

The study population was described with frequencies for qualitative variables and mean and standard deviations (SDs) for quantitative variables. For qualitative variables, groups were compared using the Chi-square test or the Fisher test. The continuous variable distributions were tested with the Shapiro–Wilk statistic. Student’s *t*-test was used to compare quantitative variables when the distribution was Gaussian, and the Mann–Whitney test was used otherwise.

Univariate and multivariate logistic regression analyses were performed to determine two risk scores for predicting the classification of patients as normal or below the expected Z-score value at lumbar spine and hip localization. Several relevant anthropometric, clinical, and disease-related parameters without collinearity were included in the model. Quantitative variables not respecting the log-linearity hypothesis were transformed using their median (for age, BMI, and highest disease-related BMI) or a receiver operating characteristic (ROC) cut-off maximizing the Youden index (for duration of amenorrhea without contraceptive). A stepwise selection based on the corrected Akaike information criterion was applied. Odds ratios (ORs) and their 95% confidence intervals (95% CIs) were calculated. Simplified risk scores were established. For each estimated parameter, tentative scores were calculated by multiplying the parameter by 10 and then rounding. To reduce the total score, each tentative score was divided by a coefficient (6 for lumbar spine score and 5 for hip score) and then rounded. To determine the discriminative ability of the score, an ROC curve analysis was used to estimate the area under the curve (AUC) with 95% CI and to establish the cut-off point optimizing the sensitivity. Bootstrap sampling with replacement was performed for 200 iterations to internally validate the simplified scores. The AUC value lies between 0.5 and 1.0, where 0.5 = no discrimination, 0.5 to 0.7 = poor discrimination, 0.7 to 0.8 = acceptable discrimination, 0.8 to 0.9 = excellent discrimination, and >0.9 = outstanding discrimination [29].

The statistical significance was set at 0.05 and analyses were conducted using Statistical Analysis Systems version 7.13 (SAS Enterprise Guide).

## 3. Results

### 3.1. Participant Characteristics

The anthropometric and clinical characteristics of the 313 patients with AN and the 121 controls are presented in Table 1. The age ranged from 14.5 to 34.9 years, with a mean age of 21.6 ± 5.2 and 21.1 ± 4.2 years for the AN group and controls, respectively. There were no significant differences between the two groups for height or age, whereas, as expected, weight, BMI, whole-body FFST, FM, and percent FM were significantly lower (*p* < 0.001) in the AN group compared with the controls. When height SDs and weight SDs were determined according to the French reference curves, AN patients presented normal values for height (0.31 ± 1.13 SD) and low values for weight (−1.66 ± 0.95 SD). In patients, the mean age of onset was 17.5 ± 3.8 (range 10.0–33.0) years, and the mean duration was 4.1 ± 4.4 (range 0.2–20.5) years. The age of menarche was not different between groups (12.9 ± 1.5 and 12.7 ± 1.5 years for AN and controls, respectively). Among the controls, only minor variations in the duration of menstrual cycles (~28 days) were encountered, but no secondary amenorrhea; whereas 69% of the patients not using OCs presented menstrual disorders. Among the patients, 34% (106 of 313) were taking OCs vs. 56% (68 of 121) of the healthy controls.

In order to build the risk score for osteoporosis, patients were next subdivided into two groups according to their lumbar spine Z-score aBMD class: 235 (75%) presented normal values (Z-score > −2 SD) and 78 (25%) presented a Z-score of −2.0 or lower, defined as the “below the expected range for age” group. The subgroup classifications according to the Z-score obtained from the lumbar spine region were close to those obtained from the hip region (245 (79%) and 66 (21%), respectively). Taking into account our classification, aBMD at the lumbar spine and hip was, as expected, lower in patients with the lowest Z-scores. Globally, the lower a patient’s Z-score was, the lower the anthropometric parameters were—including weight, BMI, WB FM, WB FFST, lowest BMI, and highest BMI—whereas the durations of AN and amenorrhea were longer and the prevalence of menstrual disorders was higher.

### 3.2. Association of Various Parameters with Z-Score at Lumbar Spine (Table 2)

In the univariate analysis, age (≥20 year vs. <20 year), BMI (<16 kg/m^2^ vs. ≥16 kg/m^2^), duration of AN, minimal disease-related BMI, menstrual disorders (no vs. yes), duration of amenorrhea without OC use (≥18 months vs. <18 months), and OC use (no vs. yes) were significantly associated with the probability of presenting aBMD below the expected range for age. Conversely, age of AN onset, highest disease-related BMI, duration of OC use, and duration of OC use during AN were not associated. In the multivariate analysis, age (≥20 year vs. <20 year), minimal disease-related BMI, and duration of amenorrhea without OC use (≥18 months vs. <18 months) remained significatively associated.

### 3.3. Association of Various Parameters with Z-Score at Hip (Table 3)

In the univariate analysis, age (≥20 year vs. <20 year), BMI (<16 kg/m^2^ vs. ≥16 kg/m^2^), duration of AN, minimal disease-related BMI, menstrual disorders (no vs. yes), duration of amenorrhea without OC use (≥18 months vs. <18 months), and OC use (no vs. yes) were significantly associated with the probability of presenting aBMD below the expected range for age. Conversely, age of AN onset, highest disease-related BMI, duration of OC use, and duration of OC use during AN were not associated. In the multivariate analysis, age (≥20 year vs. <20 year), minimal disease-related BMI, and duration of amenorrhea without OC use (≥18 months vs. <18 months) remained significatively associated.

It is interesting to note that the parameters and the odds ratios associated with the lumbar spine and hip Z-scores presented a high level of similarity.

### 3.4. Constructing the Specific Risk Score for Predicting Classification of Patients as Normal or Below the Expected Z-Score Range for Age (Figure 1)

For lumbar spine, a risk score equation was developed: [Score = 14 + 2 × Age (≥20 year vs. <20 year) − 1 × minimal disease-related BMI (kg/m^2^) + 2 × duration of amenorrhea without OC use (≥18 months vs. <18 months)] (Table 2). The AUC measuring the score’s performance was 0.85 [95% CI: 0.79–0.90] and the selected cut-off was ≥0.9, with a sensitivity of 83% and specificity of 71% (Table 4).

For hip, the risk score equation was the following: [Score = 12 + 2 × Age (≥20 year vs. <20 year) − 1 × minimal disease-related BMI (kg/m^2^) + 3 × duration of amenorrhea without OC use (≥18 months vs. <18 months)] (Table 3). Its AUC was 0.82 [95% CI: 0.76–0.86] and the selected cut-off was ≥1.33, with a sensitivity of 92% and specificity of 55% (Table 4).

Table 5 gives examples of patients with low or normal aBMD for age using the determinant cut-offs in three hypothetical patients with AN.

## 4. Discussion

The study confirmed that the patients with AN presented reduced aBMD values, with a degree of alteration that was associated with the gravity of the undernutrition identified by the BMI. Several clinical and disease-related factors have been identified as aBMD modulators, and three of them—age, minimal disease-related BMI, and duration of amenorrhea—are easily collectable in these patients and appear to be independently associated with the intensity of the bone loss. Based on these three factors, we developed and internally validated for the first time a score to identify those patients susceptible to present low bone mass for age and probably most at risk of fragility fractures.

It has been well established that women with AN present lower aBMD than normal-weight women due to an alteration in bone remodeling [3,30,31,32,33,34,35]; this may lead to an increase in the fracture risk observed in this population [36]. These findings prompted the search to develop tools for clinical practice to easily identify those patients susceptible to presenting low aBMD for age when DXA scans are not available. To our knowledge, this new approach has never been evaluated in this population.

The first step in building a risk score is to identify the factors that influence aBMD. We found that a number of factors, including age, past or present anthropometric parameters (i.e., current BMI and minimal disease-related BMI), and disease-related factors (i.e., disease duration or amenorrhea duration) were positively or negatively associated with aBMD. These results are in total agreement with previous findings [2,3,5,12,13,14,17,20,37]. Other parameters, such as whole-body lean tissue mass evaluated with DXA [2], or biological parameters such as markers of bone turnover (i.e., osteocalcin) [20], IGF-1 [17,38], adiponectin, or leptin [17] were also found to be independently associated with aBMD. Nevertheless, these parameters are often not available in clinics, which may be disadvantageous for patients. In order to develop a tool that can be used in clinical routine or by the patients themselves, only potential parameters that are easily available and that can be accurately collected were selected to design this risk score.

We identified three independent factors including age (variable in class: ≥20 year vs. <20 year), minimal disease-related BMI (continuous variable), and duration of amenorrhea (variable in class: >18 months vs. ≤18 months). It is interesting to observe that these factors were commonly present, and for a given factor the ORs were of the same order of magnitude for the two bone sites (i.e., lumbar spine and hip). These findings support the strong and constant effect of these three factors on Z-score variability. It is unsurprising that the duration of amenorrhea emerged as a determinant parameter, with the highest OR [3.68 and 4.29 in multivariate analysis], because peak bone mass acquisition during the first two decades of life, the principal period when AN occurs, is estrogen-dependent [39,40]. In young non-AN women, it is well known that hypogonadal states are characterized by low mineralization [39]. In patients with AN, the severity of the bone demineralization is negatively correlated with the duration of regular menses before amenorrhea and positively correlated with the duration of amenorrhea [3,15,22]. The detrimental effect of estrogen secretion in the adolescent period may be particularly detrimental for bone mass acquisition, as demonstrated by the greater aBMD deficiency in patients with primary amenorrhea than in patients with secondary amenorrhea and later disease onset [22]. Moreover, adult patients who develop AN during adolescence have lower aBMD than those who develop AN during adulthood, even when the duration of amenorrhea is comparable [41]. Weight gain and restoration of menstrual cycle appear to be the optimal condition to recover aBMD [1,15]. Finally, the duration of amenorrhea should probably be considered as an integrative parameter of the duration of the disease with a high degree of severity [3]. As suggested by amenorrhea duration, the bone status of patients is the result of a long process that integrates the duration and the severity of the disease. In this context, the minimal disease-related BMI, which represents the severity of underweight since the onset of AN, was independently associated with the Z-score, while the current BMI was not. This finding indicates that, to influence aBMD, a certain minimal weight during the history of the disease is required. The last independent factor is the age of the patient, which may also partly reflect the disease duration but, contrary to the amenorrhea duration, provides no information on disease severity.

The predicted model revealed an AUC of 0.82 associated with a sensitivity of 83% for the low-bone-mass-for-age diagnosis. Given the lack of another available risk score for this population, we cannot compare the performance of our model; nevertheless, the AUC equal to 0.82 indicated an excellent capability to distinguish between patients with and without low bone mass for age [29]. This model can be used to decide whether a patient should undergo a DXA examination and may also help make patients aware of the negative consequences of the disease. Moreover, it seems to present other qualities: (i) the identification of these three independent factors to build the risk score is quite fortuitous because they are all easily, precisely, and consistently collected from patients needing close medical monitoring and when body weight measurement is part of the patient’s routine. Moreover, our experience has shown that the date of the last menstrual cycle, which is needed to calculate the amenorrhea duration, is always recorded. (ii) This model has a very good capability to discriminate patients with low bone mass for age and thus those with the highest risk of developing fragility fractures, suggesting its clinical relevance [42]. (iii) The score is very easy to use in its present form, as demonstrated by its application in three hypothetical patients. Nevertheless, the development of a Web interface may improve its ease of use for the medical community or by the patients themselves. (iv) This score could improve the likelihood of a more rapid low-bone-mass-for-age diagnosis.

We are aware that our study presents some limitations: (i) our score was developed using a group of patients with specific characteristics (15–35 years), including low but not very low mean BMI, which might be observed in other studies. This may have potentially impacted the coefficients of the three factors used. Nevertheless, the constitution of a large group of patients, including those with extremely low BMI (9.3 kg/m^2^), suggested that the modifications should not be made only at the margins; (ii) moreover, although we performed an internal validation, a reliable external validation is required in another external population of patients [43]; (iii) the specificity may appear somewhat low compared to the sensitivity, but we chose to optimize the diagnosis of patients with low bone mass for age to the detriment of limiting the number of DXA exams; (iv) we must warn the user about specific conditions that may bias the diagnosis. For example, for a patient who presents withdrawal bleeding due to recent OC use after 2 years of amenorrhea, the clinician should choose the “1” value that characterizes patients with amenorrhea for > 16 months in order to take into account the long-term negative effect of amenorrhea; and (v) as mentioned above, several biological parameters [17,20,38] or physical activity may affect [22] aBMD and their inclusion in the models might improve the sensitivity of the risk score. However, these parameters are rarely available in clinical settings or should be considered with caution when data are obtained through questionnaires due to the denial that exists in this pathology [44].

## 5. Conclusions

The study presents for the first time a risk score for diagnosing low bone mass for age in young patients with AN. Taking into account its good sensitivity and its ease of use that requires only three parameters that are well identified in this disease (i.e., age, minimal lowest BMI, and duration of amenorrhea), this new score may be useful in clinical settings when DXA scans are not feasible. This risk score could enable earlier and more systematic screening for low bone mass in this AN population, and enable specific treatment to be implemented earlier. Further studies are needed to replicate these data in independent samples and on complementary populations (atypical anorexia, avoidant restrictive food intake disorders, constitutional thickness…). Longitudinal studies would also be interesting to test the score sensitivity over the course of the disease (i.e., worsening or remission).

## Figures and Tables

**Figure 1:**
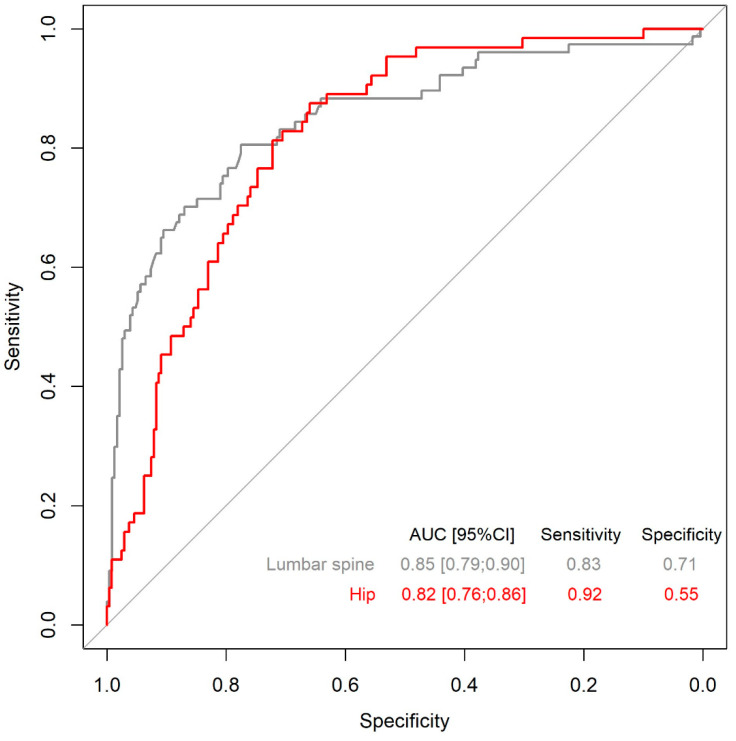
Receiver operating characteristic (ROC) curves to evaluate the discriminative ability of the score to predict low bone mineral density for age at lumbar spine and hip in patients with anorexia nervosa.

**Table 1:** Characteristics of controls and patients with anorexia nervosa (AN) according to Z-score values evaluated at lumbar spine.

Parameter	Controls	All AN Patients	Z-Score > −2 SD (Normal)	Z-Score ≤ −2 SD (Low aBMD for Age)
Number of subjects	121	313	235	78
Age (year)	21.1 ± 4.2	21.6 ± 5.2	20.6 ± 4.7	24.4 ± 5.6 *
Anthropometric data				
Weight (kg)	59.2 ± 7.6	43.1 ± 5.6 ^#^	44.2 ± 4.8	40.0 ± 6.6 *
Weight (SD)	0.94 ± 1.24	−1.66 ± 0.95 ^#^	−1.48 ± 0.79	−2.21 ± 1.16 *
Height (cm)	165.2 ± 6.0	164.4 ± 6.2	165.0 ± 6.2	162.6 ± 5.9 *
Height (SD)	0.48 ± 1.09	0.31 ± 1.13	0.43 ± 1.11	−0.06 ± 1.11 *
BMI (kg·m^−2^)	21.6 ± 2.3	15.9 ± 1.6 ^#^	16.2 ± 1.3	15.1 ± 2.1 *
WB fat mass (%)	27.8 ± 4.9	16.2 ± 5.5 ^#^	16.8 ±5.2	14.5 ± 5.9 *
WB fat mass (kg)	16.6 ± 4.7	7.3 ± 3.3 ^#^	7.7 ± 3.2	6.1 ± 3.1 *
WB fat-free soft tissue (kg)	40.5 ± 4.4	34.6 ± 4.6 ^#^	35.2 ± 4.3	32.8 ± 4.8 *
Characteristics of AN				
Age of AN onset (year)	-	17.5 ± 3.8	17.3 ± 3.5	18.1 ± 4.5
Duration of AN (year)	-	4.1 ± 4.4	2.6 ± 3.4	5.7 ± 5.2 *
Hyperactivities (n (%))	-	88 (28)	70 (30)	18 (23)
Lowest disease-related BMI (kg·m−^2^)	-	14.5 ± 1.7	15.0 ± 1.4	13.1 ± 1.9 *
Age at lowest disease-related BMI (year)	-	20.0 ± 4.7	19.4 ± 4.3	22.0 ± 5.5 *
Highest disease-related BMI (kg·m−^2^)	-	20.7 ± 3.1	20.7 ± 2.6	20.7 ± 4.2
Age at highest disease-related BMI (year)		17.6 ± 3.7	17.5 ± 3.6	17.9 ± 4.0
Gynecological profile				
Age of menarche (year)	12.7 ± 1.5	12.9 ± 1.5	12.8 ± 1.4	13.0 ± 1.6
Menstrual disorders (n (%))	17 (14) $	214 (69) ^#^	149 (64)	65 (83) *
Duration of amenorrhea (mo) ^a^	-	24.9 ± 43.8	13.9 ± 31.8	51.4 ± 56.1 *
Duration of amenorrhea without contraceptive (mo) (n (%)) ^b^				
<18 months	111(100)	250 (81)	210 (90)	40 (52) *
≥18 months	0 (0)	60 (19)	23 (10)	37 (48) *
Contraceptive used (n(%))	68 (56)	106 (34) ^#^	88 (38)	18 (23) *
Duration of contraceptive used (year)	1.9 ± 2.7	1.1 ± 2.8 ^#^	1.1 ± 2.6	1.2 ± 3.3
Duration of contraceptive during AN (year) ^c^	-	0.75 ± 1.99	0.76 ± 1.94	0.72 ± 2.13
Duration of contraceptive during AN (year) (n (%))				
Without contraceptive	-	205 (69)	145 (66)	60 (77)
[0;1] years	-	34 (11)	31 (14)	3 (4)
[1;3] years	-	36 (12)	26 (12)	10 (13)
>3 years	-	23 (8)	18 (8)	5 (6)
Areal bone mineral density				
Lumbar spine (g/cm^2^)	0.983 ± 0.106	0.872 ± 0.116 ^#^	0.918 ± 0.090	0.732 ± 0.064 *
Lumbar spine Z-score	−0.2 ± 1.0	−1.2 ± 1.1 ^#^	−0.7 ± 0.9	−2.7 ± 0.6 *
Hip (g/cm^2^)	0.944 ± 0.143	0.808 ± 0.131 ^#^	0.856 ± 0.101	0.663 ± 0.103 *
Hip Z-score	−0.1 ± 0.9	−1.2 ± 1.1 ^#^	−0.8 ± 0.8	−2.4 ± 0.9 *
Hip Z-score (n (%))				
Normal (Z-score > −2SD)		245 (79)	221 (94)	24 (31) *
Low aBMD for age (Z-score ≤ −2SD)		66 (21)	13 (6)	53 (69) *

Legend: Values are presented as mean ± standard deviation (SD). BMI: body mass index. $ Controls presented only minor alteration of duration of menstrual cycles (~28 days). ^a^ For patients without contraceptive only. ^b^ For patients with normal menstrual cycles or using oral contraceptives, the value “0” was attributed for the duration of amenorrhea. ^c^ For patients not using oral contraceptives, the value “0” was attributed for the duration of contraceptive use; for patients using oral contraceptives before the onset of anorexia nervosa, the duration of oral contraceptive use attributed was equal to the anorexia nervosa duration; for patients using oral contraceptives after the onset of anorexia nervosa, the duration of oral contraceptive use was retained. # Denotes a significant difference between patients with anorexia nervosa and controls with *p* < 0.001. * Denotes a significant difference between normal Z-score group and low-aBMD-for-age group with *p* < 0.05.

**Table 2:** Factors associated with normal- or low-areal-bone-mineral-density-for-age status defined by the Z-score at lumbar spine in patients with anorexia nervosa.

	Univariate Analysis		Multivariate Analysis (*n* = 308)		Scores for the Categories of Each Model		
Variable	OR [95% CI]	*p*-Value	OR [95% CI]	*p*-Value	Parameter Estimates by Logistic Regression	Tentative Score ^a^	Final Score ^b^
Age (≥20 year vs. <20 year)	4.58 [2.55;8.23]	<0.001	3.72 [1.86;7.44]	<0.001	1.314	13	2
BMI (<16 kg/m^2^ vs. ≥16 kg/m^2^)	2.64 [1.55;4.50]	<0.001	-		-		
Age of AN onset (year)	1.05 [0.99;1.12]	0.122	-		-		
Duration of AN (year) *	1.15 [1.09;1.22]	<0.001	-		-		
Lowest disease-related BMI (kg/m^2^)	0.47 [0.38;0.58]	<0.001	0.55 [0.44;0.68]	<0.001	−0.606	−6	−1
Highest disease-related BMI (<20 kg/m^2^ vs. ≥20 kg/m^2^)	1.26 [0.75;2.11]	0.390	-		-		
Menstrual disorders (no vs. yes)	2.85 [1.49;5.48]	0.002	-		-		
Duration of amenorrhea without contraceptive use (≥18 months vs. <18 months)	8.44 [4.54;15.71]	<0.001	3.68 [1.77;7.65]	<0.001	1.303	13	2
Contraceptive use (no vs. yes)	2.02 [1.12;3.65]	0.019	-		-		
Duration of contraceptive use (year)	1.02 [0.93;1.11]	0.732	-		-		
Duration of contraceptive use during AN (year)		0.123	-		-		
Without contraceptive use vs. >3 years	1.49 [0.53;4.20]						
[0;1] years vs. >3 years	0.35 [0.07;1.63]						
[1;3] years vs. >3 years	1.38 [0.40;4.74]						

OR: odds ratio; CI: confidence interval; BMI: body mass index; AN: anorexia nervosa. * Not included in multivariate analysis due to the high collinearity and correlation between age and duration of AN. ^a^: For each parameter estimate, multiplied by 10 times and rounded. ^b^: Tentative score divided by 6 and rounded.

**Table 3:** Factors associated with normal- or low-areal-bone-mineral-density-for-age status defined by the Z-score at hip in patients with anorexia nervosa.

	Univariate Analysis		Multivariate Analysis (*n* = 305)		Scores for the Categories of Each Model		
Variable	OR [95% CI]	*p*-Value	OR [95% CI]	*p*-Value	Parameter Estimates by Logistic Regression	Tentative Score ^a^	Final Score ^b^
Age (≥20 year vs. < 20 year)	3.27 [1.80;5.95]	<0.001	2.34 [1.17;4.69]	0.017	0.850	9	2
BMI (<16 kg/m^2^ vs. ≥16 kg/m^2^)	2.71 [1.53;4.80]	<0.001	-		-		
Age of AN onset (year)	1.01 [0.94;1.08]	0.882	-		-		
Duration of AN (year) *	1.18 [1.11;1.25]	<0.001	-		-		
Lowest disease-related BMI (kg/m^2^)	0.51 [0.42;0.62]	<0.001	0.61 [0.49;0.75]	<0.001	−0.500	−5	−1
Highest disease-related BMI (<20 kg/m^2^ vs. ≥20 kg/m^2^)	1.69 [0.96;2.98]	0.067	-		-		
Menstrual disorders (no vs. yes)	2.41 [1.22;4.74]	0.011	-		-		
Duration of amenorrhea without contraceptive use (≥18 months vs. <18 months)	9.15 [4.85;17.26]	<0.001	4.29 [2.09;8.80]	<0.001	1.457	15	3
Contraceptive use (no vs. yes)	2.17 [1.14;4.13]	0.019	-		-		
Duration of contraceptive use (year)	1.01 [0.91;1.11]	0.858	-		-		
Duration of contraceptive use during AN (year)		0.128	-		-		
Without contraceptive use vs. >3 years	1.20 [0.42;3.40]						
[0;1] years vs. >3 years	0.23 [0.04;1.32]						
[1;3] years vs. >3 years	0.72 [0.19;2.70]						

OR: odds ratio; CI: confidence interval; BMI: body mass index; AN: anorexia nervosa. * Not included in multivariate analysis due to the high collinearity and correlation between age and duration of AN. ^a^: For each parameter estimate, multiplied by 10 times and rounded. ^b^: Tentative score divided by 5 and rounded.

**Table 4:** ROC analysis—performance of scores.

	AUC [95% CI] *	Cut-Off ^a^	Sensitivity	Specificity	TP	TN	FP	FN	PPV	NPV
Lumbar spine	0.85 [0.79;0.90]	0.9	0.83	0.71	64	164	67	13	0.49	0.93
Hip	0.82 [0.76;0.86]	1.33	0.92	0.55	59	134	107	5	0.36	0.96

*: Internally valid (bootstrap). ^a^: diagnosis of low-aBMD-for-age status if score ≥ cut-off. TP: true positive; TN: true negative; FP: false positive; FN: false negative; PPV: positive predictive value; NPV: negative predictive value.

**Table 5:** Diagnosis of low-aBMD-for-age status using determined cut-offs in three hypothetical patients with anorexia nervosa.

**Risk score for low BMD in lumbar spine. Calculation of score: 14** **—lowest disease-related BMI + 2 if subject is over 20 + 2 if subject presents amenorrhea for more than 18 months. A net score greater than 0.9 indicates a risk of low aBMD for age.** **Risk score for low BMD in hip. Calculation of score: 12—lowest lifetime BMI + 2 if subject is over 20 + 3 if subject presents amenorrhea for more than 18 months. A net score greater than 1.33 indicates a risk of low aBMD.**				
		**Patient 1**	**Patient 2**	**Patient 3**
Age (year) ^a^		22	20	20
Lowest disease-related BMI (kg/m^2^) ^b^		10	16.5	12
Duration of amenorrhea without contraceptive use (months) ^c^		48	0	24
Lumbar spine	Score ^d^	14 − 10 + 2 + 2 = 8	14 − 16.5 + 2 = −0.5	14 − 12 + 2 + 2 = 6
	Diagnosis of low-aBMD-for-age status (if ≥0.9)	Low aBMD for age	Normal	Low aBMD for age
Hip	Score ^e^	12 − 10 + 2 + 3 = 7	12 − 16.5 + 2 = −2.5	12 − 12 + 2 + 3 = 5
	Diagnosis of low-aBMD-for-age status (if ≥1.33)	Low aBMD for age	Normal	Low aBMD for age

^a^: For age: value “0” was attributed for age < 20 year and value “2” for age ≥ 20 year. ^b^: For lowest BMI, the continuous variable was used. ^c^: For duration of amenorrhea without contraceptive: value “0” was attributed for duration < 18 months or if patient used oral contraceptives, and values “2” for spine and “3” for hip were attributed for duration ≥ 18 months. ^d^: To calculate the score, the constant (intercept) 14 has to be added to the equation. ^e^: To calculate the score, the constant (intercept) 12 has to be added to the equation.

## Data Availability

The data used in the present analysis can be obtained through request to the corresponding author.
